# Genetic Diversity of Shanlan Upland Rice (*Oryza sativa* L.) and Association Analysis of SSR Markers Linked to Agronomic Traits

**DOI:** 10.1155/2021/7588652

**Published:** 2021-10-19

**Authors:** Guofeng Yang, Yong Yang, Yali Guan, Zhixia Xu, Junyu Wang, Yong Yun, Xiaowei Yan, Qingjie Tang

**Affiliations:** ^1^Key Laboratory of Tropical Animal and Plant Ecology of Hainan Province, College of Life Sciences, Hainan Normal University, Haikou 571158, China; ^2^Cereal Crops Institute, Sanya Institute, Hainan Academy of Agricultural Sciences, Key Laboratory of Crop Genetics and Breeding of Hainan Province, Scientific Observation and Experiment Station of Gene Resources and Germplasm Creation in Hainan, Ministry of Agriculture and Rural Affairs, Haikou 571100, China

## Abstract

Shanlan upland rice, a kind of unique rice germplasm in Hainan Island, was used to evaluate genetic diversity and association between SSR markers and agronomic traits. A total of 239 alleles were detected in 57 Hainan upland rice varieties using 35 SSR markers, and the number of alleles per locus was 2-19. The observed heterozygosity was 0.0655-0.3115. The Shannon diversity index was 0.1352-0.4827. The genetic similarity coefficient was 0.6736-0.9707, and 46 varieties were clustered into one group, indicating that the genetic base of the Shanlan upland rice germplasm was narrow. A total of 25 SSR markers significantly related to plant height, effective panicle number per plant, panicle length, total grain number, filled grain number, seed rating rate, and 1000-grain weight were obtained (*P* < 0.01), with the percentage of the total variations explained ranging from 0.12% to 42.62%. RM208 explained 42.62% of the total variations in plant height of Shanlan upland rice. RM493 was significantly associated with 6 agronomic traits. We can speculate that RM208 may flank QTLs responsible for plant height and RM493 may flank QTLs playing a fundamental role in the intertwined regulatory network of agronomic traits of Shanlan upland rice.

## 1. Introduction

Shanlan upland rice (*Oryza sativa* L.) is a type of landrace adapted to the tropical dryland climate, with strong drought resistance and good taste quality [1–3], distributed in the central and western regions of Hainan Island where Li and Miao people live. Shanlan liquor made from Shanlan glutinous rice is known as “Moutai of Li people” and well received by local people. In our previous study, the coefficients of variation of the agronomic traits including plant height, panicle length, seed setting rate, and 1000-grain weight were all more than 10%, and the coefficients of variation of the traits including effective panicle number per plant, total number of grains, and number of filled grains reached about 30%, indicating that the Shanlan upland rice germplasm has a relatively rich diversity of agronomic traits. Many excellent Shanlan upland rice varieties were found out, including 7 varieties with panicle length exceeding 30.0 cm, 3 varieties with total number of grains being more than 200.0, 10 varieties with seed setting rate exceeding 90.0%, and 23 varieties with 1000-grain weight being more than 30.0 g, which would provide excellent genetic resources for the high-yield breeding of rice [4].

Based on linkage disequilibrium (LD), the association between target traits and genetic markers or candidate gene mutations in the natural population can be identified. It is widely used in association analysis between molecular and phenotypic variation, and discovery, location, and functional analysis of genes of interest [5–7]. Simple sequence repeat (SSR) is composed of a set of 1 to 6 base sequences (motif) repeated in tandem, with high polymorphism, abundant quantity, good repeatability, and codominance [8, 9]. The Gramene website (http://www.gramene.org) has published more than 19,000 SSR markers in rice, which are commonly used in rice genetic diversity studies, germplasm evaluation, genetic map construction, target trait gene location, and cloning.

In previous studies, only few Shanlan upland rice varieties were used in the analyses of the genetic diversity [1, 10]. No report on association analysis between SSR markers and agronomic traits of the Shanlan upland rice germplasm was found. The objectives of this present study were to use 57 Shanlan upland rice varieties to evaluate genetic diversity and analyze association between SSR markers and agronomic traits.

## 2. Materials and Methods

### 2.1. Materials and Experimental Site

A collection consisting of 57 Shanlan upland rice accessions was used in this study, which including landraces and other breeding varieties collected from the central and western regions of Hainan Island during 2013–2017 (see Table 1). From 2015 to 2017, all accessions were grown in the Yongfa Base of Hainan Academy of Agricultural Sciences in Chengmai County, Hainan Province, and Tropical Crop Field in Yinggen Town, Qiongzhong County, Hainan Province, and were planted around the Dragon Boat Festival in a direct-seeding way, with shallow soil cover. 100 plants of each accession were planted with 25 cm of row spacing and 30 cm of plant spacing. The conventional management of upland rice planting was performed.

### 2.2. Agronomic and Molecular Methods in the Study

Ten plants were selected at random from each accession and evaluated for 7 agronomic traits including plant height, effective panicle number per plant, panicle length, total number of grains per panicle, number of filled grains per panicle, seed setting rate, and 1000-grain weight. Analysis of variance (ANOVA) was performed using SPSS 19.0 software.

A total of 48 SSR markers distributed on 12 chromosomes of rice were used to survey the Shanlan upland rice germplasm for genetic diversity (see Table 2). The forward fluorescent primers of SSR markers were filled with FAM (blue) fluorescent dye, and synthesized by the BGI (Guangzhou) Company. The reverse nonfluorescent primers were synthesized by the Shenggong (Shanghai) Company.

Using the Plant Genomic DNA Rapid Extraction Kit produced by the Shenggong (Shanghai) Company, the genomic DNA was extracted from 50 to 100 mg of the fresh tender leaves collected and sampled from individual plants of the accessions at the seedling stage as a template. The PCR reactions were carried out in a reaction solution of 10 *μ*L containing 1 *μ*L of the template DNA (50~100 ng), 0.2 *μ*L of each primer (10 *μ*mol/L), 5 *μ*L of the 2x EasyTaq® PCR SuperMix produced by TransGen Biotech Company, and 3.6 *μ*L of ddH_2_O. The PCR amplification reactions were performed at the following cycle profile: initial denaturation at 94°C for 4 min, 30 cycles of 45 s denaturation at 94°C, 45 s annealing at 50~67°C, 1 min extension at 72°C, followed by 8 min at 72°C for the final extension. The amplified products were submitted to the BGI (Wuhan) Company for capillary electrophoresis by the ABI 3730xl Genetic Analyzer, and the original data was collected using Data Collection software.

A 1/0 matrix was constructed based on the presence and absence of alleles. The presence was denoted as 1 and absence as 0. The genetic diversity parameters such as number of alleles per locus, observed heterozygosity (Ho), and Shannon's diversity index (*I*) were estimated using the program Popgene 1.32. The genetic similarity coefficients among the accessions were calculated using NTSYS 2.1 software. The cluster analysis was carried out usying the UPGMA and SHAN methods. The population structure was estimated using Structure 2.2 software.

Association analyses were carried out using Tassel 2.1 software. The maximum of *L*(*K*) was identified as the optimum number of the subpopulation, and the structure matrix (*Q*) was extracted from the membership probability of each genotype for the mixed linear model (MLM) analysis.

## 3. Results

### 3.1. Genetic Diversity Analysis

**Figure 1:**
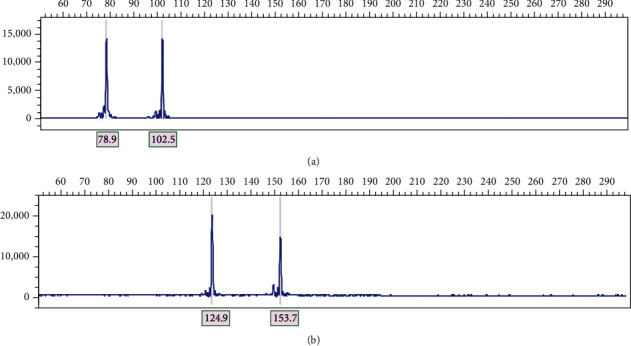
Allele report based on capillary electrophoresis. (a) SSR marker RM85 was used to survey Shanlan upland rice accession M36. Two alleles were detected and denoted as 79 and 103. (b) SSR marker RM336 was used to survey Shanlan upland rice accession M31. Two alleles were detected and denoted as 125 and 154.

35 polymorphic SSR markers selected from a total of 48 SSR markers were used to screen 57 Shanlan upland rice accessions. As shown in Table 3, a total of 239 alleles were detected. A couple of allele report images are shown in Figure 1. The number of alleles per locus varied from 2 to 19 with an average of 6.8. The observed heterozygosity ranged from 0.0655 to 0.3115 with an average of 0.1702. The Shannon diversity index ranged from 0.1352 to 0.4827 with an average of 0.2826.

### 3.2. Genetic Similarity Coefficient and Cluster Analysis

**Figure 2:**
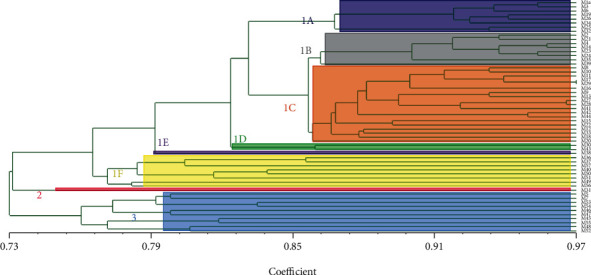
Neighbor-joining cluster analysis for Shanlan upland rice accessions. Subclade 1A: M1, M3, M6, M19, M26, M34, M25, and M32. Subclade 1B: M2, M21, M4, M14, M23, M24, M35, and M39. Subclade 1C: M8, M10, M11, M17, M29, M16, M9, M13, M27, M28, M41, M42, M44, M15, M22, M12, M33, M18, and M20. Subclade 1D: M30 and M43. Subclade 1E: M38. Subclade 1F: M36, M37, M57, M40, M50, M51, M49, and M56. Clade 2: M31. Clade 3: M5, M7, M53, M54, M46, M47, M45, M55, M48, and M52.

The genetic similarity coefficients of 57 Shanlan upland rice accessions ranged from 0.6736 to 0.9707 with an average of 0.7889. The dendrogram resulting from the distance-based analysis of 57 accessions with Jaccard's genetic distance is shown in Figure 2. 57 accessions were classified into 3 clades with a genetic similarity coefficient of 0.75. Clade 1 included 46 accessions, such as M1, M3, and M6, which were classified into 6 subclades. The accession M31 constituted Clade 2 alone. Clade 3 included 10 accessions, such as M5, M7, and M53.

### 3.3. Population Structure Analysis

**Figure 3:**
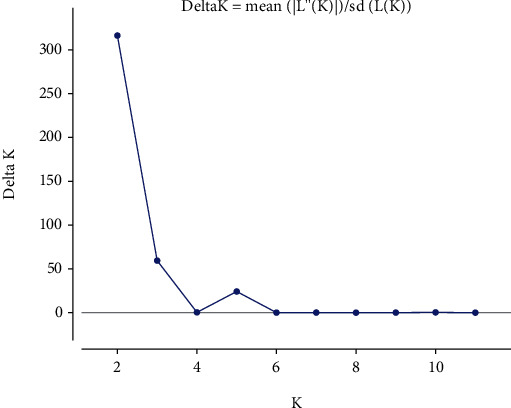
Estimation of population in Shanlan upland rice accessions.

**Figure 4:**
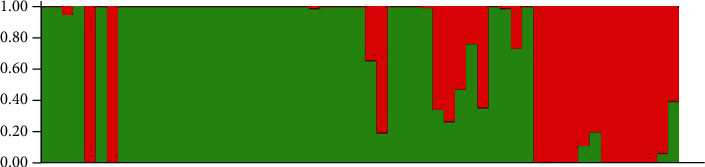
Population structure of Shanlan upland rice accessions.

The likelihood value of this analysis is shown in Figure 3. The likelihood was maximum at 2 of *K* value and then decreased, after which it became almost constant. Therefore, the structure results of *K* = 2 were considered the best possible partition. Using Structure 2.2 software, the posterior probability of each accession was calculated, and 57 Shanlan upland rice accessions were divided into 2 subpopulations. The population structure diagram is shown in Figure 4. Subpopulation 1 included 37 accessions: M1, M2, M3, M4, M6, M8, M9, M10, M11, M12, M13, M14, M15, M16, M17, M18, M19, M20, M21, M22, M23, M24, M25, M26, M27, M28, M29, M30, M32, M33, M34, M35, M39, M41, M42, M43, and M44. Subpopulation 2 included 20 accessions: M5, M7, M31, M36, M37, M38, M40, M45, M46, M47, M48, M49, M50, M51, M52, M53, M54, M55, M56, and M57.

Comparing the results of population structure analysis with cluster analysis, it was found that the accessions contained in Subpopulation 1 were consistent with those contained in Subclade 1A, Subclade 1B, Subclade 1C, and Subclade 1D. Subclade 1E, Subclade 1F, Clade 2, and Clade 3 belonged to Subpopulation 2.

### 3.4. Association Analysis

The details of the agronomic traits of 57 Shanlan upland rice accessions are shown in Table 4. The analysis of variance revealed significant differences (*P* < 0.05) among 57 accessions for 7 agronomic traits, respectively.

**Figure 5:**
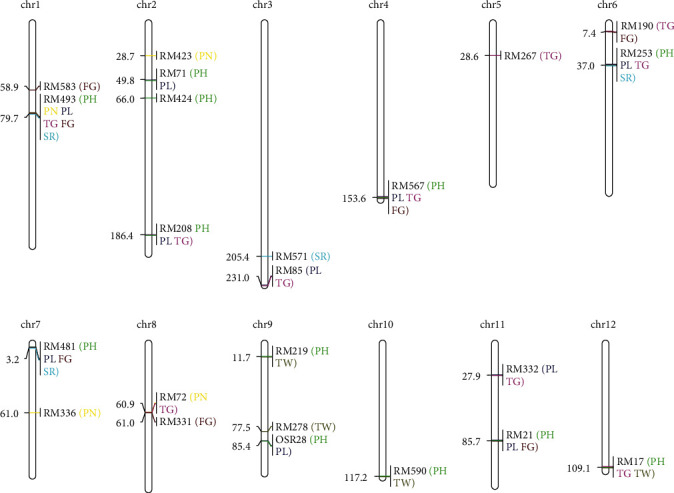
Map locations of the SSR markers linked to agronomic traits of Shanlan upland rice accessions. PH: plant height. PN: effective panicle number per plant. PL: panicle length. TG: total number of grains. FG: number of filled grains. SR: seed setting rate. TW: 1000-grain weight.

A total of 25 SSR markers significantly associated with agronomic traits such as plant height, effective panicle number per plant, panicle length, total grain number, filled grain number, seed setting rate, and 1000-grain weight were detected, with the percentage of total variations explained ranging from 0.12% to 42.62% (*P* < 0.01) (see Table 5). Of them, RM208 explained 42.62% of total variations in plant height of Shanlan upland rice. The locations of the associated SSR markers on 12 chromosomes of rice are shown in Figure 5 according to Cornell SSR 2001 (https://archive.gramene.org/). The SSR markers significantly associated with each agronomic trait of the Shanlan upland rice germplasm were all distributed on multiple chromosomes. There were many SSR markers significantly linked to 2 or more agronomic traits, respectively. For example, 8 markers were significantly associated with 2 traits, 3 markers with 3 traits, and 3 markers with 4 traits. In particular, RM493 was significantly associated with 6 traits.

## 4. Discussion

Shanlan upland rice is a kind of unique rice germplasm in Hainan Island. So, it is essential to analyze its genetic diversity and explore its application in rice breeding. Through the sequence analysis of *SSII*, *ITS*, *Ehd1*, *ndhC-trnV*, and *cox3* genes, Yuan et al. have found that the genetic diversity of 14 Shanlan upland rice varieties was lower than that of Asian cultivated rice and the common wild rice [1]. Wang et al. have analyzed the genetic diversity of 23 Shanlan upland rice varieties using 22 RAPD primers and found that the genetic similarity coefficients ranged from 0.881 to 0.952 [10]. In this study, a total of 239 alleles were detected in 57 Hainan upland rice varieties using 35 SSR markers, and the number of alleles per locus varied from 2 to 19 with an average of 6.8. The genetic similarity coefficient of 57 Shanlan upland rice varieties ranged from 0.6736 to 0.9707 with an average of 0.7889, and 46 varieties were clustered into one group, indicating that the genetic base of the Shanlan upland rice germplasm is narrow, which is similar to the results of previous studies [1, 10]. The low genetic diversity of the Shanlan upland rice germplasm may be caused by factors such as the relatively single geographic origin and the long-term continuous selection of Li and Miao people.

In this study, 57 accessions were classified into 3 clades, and Clade 1 was further classified into 6 subclades through cluster analysis. Through population structure analysis, 57 accessions were divided into 2 subpopulations. Subclade 1A, Subclade 1B, Subclade 1C, and Subclade 1D belonged to Subpopulation 1. Subclade 1E, Subclade 1F, Clade 2, and Clade 3 belonged to Subpopulation 2. Subclade 1A included 8 accessions, all of which belonged to the *japonica* subspecies except for M34. Subclade 1B included 8 accessions, all of which belonged to the *japonica* subspecies except for M14. Subclade 1C included 19 accessions, of which 8 accessions belonged to the *japonica* subspecies and 11 accessions belonged to the *indica* subspecies. Subclade 1D included 2 accessions, one of which belonged to the *japonica* subspecies and the other belonged to the *indica* subspecies. Subclade 1E included 1 accession, which belonged to the *japonica* subspecies. Subclade 1F included 8 accessions, of which 5 accessions belonged to the *japonica* subspecies and 3 accessions belonged to the *indica* subspecies. Clade 2 included 1 accession, which belonged to the *indica* subspecies. Clade 3 included 10 accessions, of which 4 accessions belonged to the *japonica* subspecies and 6 accessions belonged to the *indica* subspecies. Most of the accessions of some subclades belonged to the *japonica* subspecies. However, the *indica* subspecies and the *japonica* subspecies were mixed in most clades and subclades. From the perspective of geographic origin, clades and subclades were not concentratedly distributed, and the difference in their geographic origin was not obvious. Zheng et al. have used five indicators such as glume hair, grain phenol reaction, 1 to 2 internode length below the spike, grain length/width ratio, and chaff color at heading to detect the species margin of Shanlan upland rice accessions and found that most of the accessions belong to the *japonica* subspecies [11]. We can speculate that the subspecies structure of Shanlan upland rice has changed in the past two decades, and the proportion of the *indica* subspecies has increased. The area of Hainan Island is not large, and the geographical and climatic conditions, such as altitude, temperature, and sunshine, are very similar in the areas where Shanlan upland rice is cultivated. In addition, the frequent exchanges between the Li and Miao ethnic groups have made the geographical boundaries of different Shanlan upland rice accessions increasingly blurred.

Rice agronomic traits are mostly quantitative traits, controlled by multiple genes. Known rice plant height QTLs exist on each chromosome of rice, and the mapping results and the effect value of the same QTL in different studies are different [12]. Currently, nearly 90 rice dwarf genes have been discovered, and most of them are phytohormone biosynthesis defective mutations or signal transduction defective mutations [13]. Liu et al. have cloned the *THIS1* gene on rice chromosome 1 that affects tillering of rice [14]. Jiao et al. have mapped the rice tiller number QTL on chromosome 8, and finally cloned the *IPA1* (*WFP*) gene that affects tillering of rice [15]. Yan et al. and Kong et al. have mapped the rice erect panicle QTL between RM3700 and RM7424 on chromosome 9, and then Huang et al. cloned the *DEP1* gene, the mutation of which enhances the vigor of meristems and leads to the lengthening of internodes of inflorescence, the increase of panicle length and number of grains per panicle [16–18]. Ashikari et al. have cloned the *Gn1a* gene that controls the number of grains per panicle from rice chromosome 1. When *Gn1a* expression decreases, cytokinins would accumulate in the inflorescence meristems, thereby increasing the number of reproductive organs and grains per panicle [19]. Rice grain weight is greatly affected by grain shape. *GW2*, *GS3*, *GL3*, and other genes are the major genes that control rice grain weight [20–22].

In this study, a total of 25 SSR markers significantly related to plant height, effective panicle number per plant, panicle length, total grain number, filled grain number, seed rating rate, and 1000-grain weight were obtained (*P* < 0.01), with the percentage of total variations explained ranging from 0.12% to 42.62%. 12 SSR markers distributed on 9 chromosomes were significantly associated with plant height. RM208 explained 42.62% of the total variations in plant height. We can speculate that RM208 may flank QTLs responsible for plant height. Four SSR markers distributed on chromosomes 1, 2, 7, and 8 were significantly associated with the effective panicle number per plant that were similar to the results of Liu et al. and Jiao et al. partly [14, 15]. 10 SSR markers distributed on 8 chromosomes were significantly associated with panicle length. 15 SSR markers distributed on 10 chromosomes were significantly associated with the total number of grains or the number of filled grains. Five SSR markers distributed on chromosomes 9, 10, and 12 were significantly associated to 1000-seed weight. No marker associated with 1000-grain weight mentioned by previous reports was found on chromosomes 2 and 3 [20–22]. It may be related to the germplasm specificity of Shanlan upland rice and needs further study.

In this study, the SSR markers associated with each agronomic trait of Shanlan upland rice were all distributed on multiple chromosomes, which proves to a certain extent that these agronomic traits were regulated by multiple genes. On the other hand, this study also found that many SSR markers were significantly associated with 2 or more agronomic traits. RM493 was significantly associated with 6 agronomic traits. We can speculate that RM493 may flank QTLs playing a fundamental role in the intertwined regulatory network of agronomic traits of Shanlan upland rice. The genes encoding protein GFS12 (LOC4326972), protein transport protein SEC23 (LOC4326988), and probable 2-oxoglutarate-dependent dioxygenase AOP1.2 (LOC112939546), which may play a role in the regulation of the agronomic traits of Shanlan upland rice, are located in chromosome 1 close to RM493 [23–25]. These genes are worthy of further study.

## 5. Conclusion

A total of 239 alleles were detected in 57 Hainan upland rice varieties using 35 SSR markers, and the number of alleles per locus was 2-19. The observed heterozygosity was 0.0655-0.3115. The Shannon diversity index was 0.1352-0.4827. The genetic similarity coefficient was 0.6736-0.9707, and 46 varieties were clustered into one group, indicating that the genetic base of the Shanlan upland rice germplasm was narrow. A total of 25 SSR markers significantly related to plant height, effective panicle number per plant, panicle length, total grain number, filled grain number, seed rating rate, and 1000-grain weight were obtained (*P* < 0.01), with the percentage of the total variations explained ranging from 0.12% to 42.62%. RM208 explained 42.62% of total variations in plant height of Shanlan upland rice. RM493 was significantly associated with 6 agronomic traits. We can speculate that RM208 may flank QTLs responsible for plant height and RM493 may flank QTLs playing a fundamental role in the intertwined regulatory network of the agronomic traits of Shanlan upland rice.

## Figures and Tables

**Table 1:** Source and subspecies of 57 Shanlan upland rice accessions.

No.	Source	Subspecies	No.	Source	Subspecies
M1	Qiongzhong County	*Japonica*	M30	Qiongzhong County	*Indica*
M2	Qiongzhong County	*Japonica*	M31	Qiongzhong County	*Indica*
M3	Baisha County	*Japonica*	M32	Qiongzhong County	*Japonica*
M4	Qiongzhong County	*Japonica*	M33	Qiongzhong County	*Indica*
M5	Baisha County	*Indica*	M34	Qiongzhong County	*Indica*
M6	Baisha County	*Japonica*	M35	Wuzhishan City	*Japonica*
M7	Qiongzhong County	*Japonica*	M36	Wuzhishan City	*Japonica*
M8	Qiongzhong County	*Indica*	M37	Wuzhishan City	*Japonica*
M9	Qiongzhong County	*Japonica*	M38	Ledong County	*Japonica*
M10	Qiongzhong County	*Indica*	M39	Ledong County	*Japonica*
M11	Qiongzhong County	*Indica*	M40	Ledong County	*Japonica*
M12	Qiongzhong County	*Indica*	M41	Ledong County	*Indica*
M13	Qiongzhong County	*Japonica*	M42	Ledong County	*Japonica*
M14	Qiongzhong County	*Indica*	M43	Ledong County	*Japonica*
M15	Qiongzhong County	*Japonica*	M44	Ledong County	*Japonica*
M16	Qiongzhong County	*Japonica*	M45	Dongfang City	*Indica*
M17	Qiongzhong County	*Indica*	M46	Dongfang City	*Indica*
M18	Baisha County	*Indica*	M47	Dongfang City	*Indica*
M19	Qiongzhong County	*Japonica*	M48	Dongfang City	*Indica*
M20	Qiongzhong County	*Japonica*	M49	Dongfang City	*Indica*
M21	Qiongzhong County	*Japonica*	M50	Dongfang City	*Indica*
M22	Baisha County	*Japonica*	M51	Dongfang City	*Indica*
M23	Qiongzhong County	*Japonica*	M52	Dongfang city	*Indica*
M24	Qiongzhong County	*Japonica*	M53	Baoting County	*Japonica*
M25	Ledong County	*Japonica*	M54	Baoting County	*Japonica*
M26	Ledong County	*Japonica*	M55	Baoting County	*Japonica*
M27	Baisha County	*Indica*	M56	Ledong County	*Japonica*
M28	Baisha County	*Indica*	M57	Ledong County	*Japonica*
M29	Qiongzhong County	*Indica*			

**Table 2:** SSR markers used in study.

No.	SSR marker	Sequence (5′ to 3′)	Annealing temperature (°C)	Size of common polymorphic fragments (bp)
1	RM583	Forward: agatccatccctgtggagag Reverse: gcgaactcgcgttgtaatc	55	179, 189, 192, 195, 198
2	RM7l	Forward: ctagaggcgaaaacgagatg Reverse: gggtgggcgaggtaataatg	55	121, 139, 148, 213
3	RM85	Forward: ccaaagatgaaacctggattg Reverse: gcacaaggtgagcagtcc	55	79, 94, 100, 103
4	RM471	Forward: acgcacaagcagatgatgag Reverse: gggagaagacgaatgtttgc	55	104, 106, 114
5	RM274	Forward: cctcgcttatgagagcttcg Reverse: cttctccatcactcccatgg	55	149, 161
6	RM190	Forward: ctttgtctatctcaagacac Reverse: ttgcagatgttcttcctgatg	55	107, 119, 121
7	RM336	Forward: cttacagagaaacggcatcg Reverse: gctggtttgtttcaggttcg	55	141, 144, 151, 154, 160, 163, 165, 192
8	RM72	Forward: ccggcgataaaacaatgag Reverse: gcatcggtcctaactaaggg	55	149, 159, 162, 165
9	RM2l9	Forward: cgtcggatgatgtaaagcct Reverse: catatcggcattcgcctg	55	194, 196, 215, 221
10	RM311	Forward: tggtagtataggtactaaacat Reverse: tcctatacacatacaaacatac	55	160, 166, 170, 182
11	RM209	Forward: atatgagttgctgtcgtgcg Reverse: caacttgcatcctcccctcc	55	125, 132, 151, 153, 160
12	RM19	Forward: caaaaacagagcagatgac Reverse: ctcaagatggacgccaaga	55	216, 247, 250, 253
13	RM1195	Forward: atggaccacaaacgaccttc Reverse: cgactcccttgttcttctgg	55	142, 144, 146, 148, 150, 152
14	RM208	Forward: tctgcaagccttgtctgatg Reverse: taagtcgatcattgtgtggacc	55	162, 164, 172, 176, 178
15	RM232	Forward: ccggtatccttcgatattgc Reverse: ccgacttltcctcclgacg	55	141, 150, 156, 159, 161
16	RM119	Forward: catccccctgctgctgctgctg Reverse: cgccggatgtgtgggactagcg	67	166, 169
17	RM267	Forward: tgcagacatagagaaggaagtg Reverse: agcaacagcacaacttgatg	55	136, 138, 153, 155
18	RM253	Forward: tccttcaagagtgcaaaacc Reverse: gcattgtcatgtcgaagcc	55	213, 245
19	RM481	Forward: tagctagccgattgaatggc Reverse: ctccacctcctatgttgttg	55	135, 138, 141, 144, 147, 177, 188
20	RM339	Forward: gtaatcgatgctgtgggaag Reverse: gagtcatgtgatagccgatatg	55	140, 146, 158
21	RM278	Forward: gtagtgagcctaacaataatc Reverse: tcaactcagcatctctgtcc	55	59, 139, 141, 143
22	RM258	Forward: tgctgtatgtagctcgcacc Reverse: tggcctttaaagctgtcgc	55	128, 132, 136, 146
23	RM224	Forward: atcgatcgatcttcacgagg Reverse: tgctataaaaggcattcggg	55	120, 128, 131, 143, 153, 155, 157
24	RM17	Forward: tgccctgttattttcttctctc Reverse: ggtgatcctttcccatttca	55	153, 158, 182, 184
25	RM493	Forward: tagctccaacaggatcgacc Reverse: gtacgtaaacgcggaaggtg	55	221, 236, 245
26	RM561	Forward: gagctgttttggactacggc Reverse: gagtagctttctcccacccc	55	60, 185, 187, 193, 268, 270
27	RM8277	Forward: agcacaagtaggtgcatttc Reverse: atttgcctgtgatgtaatagc	55	165, 187, 190, 193, 196
28	RM551	Forward: agcccagactagcatgattg Reverse: gaaggcgagaaggatcacag	55	182, 184, 186, 188, 192, 194, 196
29	RM598	Forward: gaatcgcacacgtgatgaac Reverse: atgcgactgatcggtactcc	55	153, 156, 162
30	RMl76	Forward: cggctcccgctacgacgtctcc Reverse: agcgatgcgctggaagaggtgc	67	133, 136
31	RM432	Forward: ttctgtctcacgctggattg Reverse: agctgcgtacgtgatgaatg	55	166, 178, 186
32	RM331	Forward: gaaccagaggacaaaaatgc Reverse: catcatacatttgcagccag	55	63, 92, 150, 152, 158, 172
33	OSR28	Forward: agcagctatagcttagctgg Reverse: actgcacatgagcagagaca	55	136, 174, 177, 180, 183
34	RM590	Forward: catctccgctctccatgc Reverse: ggagttggggtcttgttcg	55	136, 143
35	RM21	Forward: acagtattccgtaggcacgg Reverse: gctccatgagggtggtagag	55	128, 132, 147, 158, 160
36	RM3331	Forward: cctcctccatgagctaatgc Reverse: aggaggagcggatttctctc	50	110, 122, 124, 126
37	RM443	Forward: gatggttttcatcggctacg Reverse: agtcccagaatgtcgtttcg	55	115, 119, 121, 123
38	RM490	Forward: atctgcacactgcaaacacc Reverse: agcaagcagtgctttcagag	55	93, 99, 103
39	RM424	Forward: tttgtggctcaccagttgag Reverse: tggcgcattcatgtcatc	55	240, 277, 280
40	RM423	Forward: agcacccatgccttatgttg Reverse: cctttttcagtagccctccc	55	269, 286, 293, 299
41	RM571	Forward: ggaggtgaaagcgaatcatg Reverse: cctgctgctctttcatcagc	55	54, 180, 189, 191
42	RM231	Forward: ccagattatttcctgaggtc Reverse: cacttgcatagttctgcattg	55	178, 180, 182, 184, 186, 188
43	RM567	Forward: atcagggaaatcctgaaggg Reverse: ggaaggagcaatcaccactg	55	244, 246, 248, 250, 252
44	RM289	Forward: ttccatggcacacaagcc Reverse: ctgtgcacgaacttccaaag	55	87, 106
45	RM542	Forward: tgaatcaagcccctcactac Reverse: ctgcaacgagtaaggcagag	55	87, 89, 111
46	RM316	Forward: ctagttgggcatacgatggc Reverse: acgcttatatgttacgtcaac	55	165, 182, 184, 186, 196, 198, 200, 213
47	RM332	Forward: gcgaaggcgaaggtgaag Reverse: catgagtgatctcactcaccc	55	164, 167, 169, 176
48	RM7102	Forward: taggagtgtttagagtgcca Reverse: tcggtttgcttatacatcag	55	170, 175, 187

**Table 3:** Genetic diversity parameters of SSR loci used for genotyping in Shanlan upland rice accessions.

No.	SSR loci	Number of alleles	Observed heterozygosity	Shannon's diversity index
1	RM583	5.0	0.1855	0.3234
2	RM490	7.0	0.1378	0.2304
3	RM443	4.0	0.3115	0.4827
4	RM493	8.0	0.1032	0.1793
5	RM424	6.0	0.1270	0.2177
6	RM423	5.0	0.1999	0.3399
7	RM561	7.0	0.2134	0.3524
8	RM208	11.0	0.0847	0.1554
9	RM71	5.0	0.1629	0.2699
10	RM231	7.0	0.2015	0.3357
11	RM571	6.0	0.2269	0.3582
12	RM8277	6.0	0.1683	0.2887
13	RM85	4.0	0.1884	0.3094
14	RM567	9.0	0.1461	0.2425
15	RM551	10.0	0.1409	0.2473
16	RM471	3.0	0.2688	0.4192
17	RM274	2.0	0.2948	0.4573
18	RM267	5.0	0.1944	0.3270
19	RM190	5.0	0.1845	0.2956
20	RM253	5.0	0.1584	0.2498
21	RM432	4.0	0.2004	0.3204
22	RM481	19.0	0.0655	0.1352
23	RM336	13.0	0.0797	0.1608
24	RM331	7.0	0.1889	0.3235
25	RM72	5.0	0.1867	0.3151
26	RM316	10.0	0.2073	0.3366
27	OSR28	8.0	0.1231	0.2174
28	RM278	6.0	0.2505	0.3817
29	RM219	7.0	0.1322	0.2273
30	RM590	5.0	0.1482	0.2430
31	RM332	6.0	0.1448	0.2467
32	RM21	10.0	0.0935	0.1681
33	RM7102	5.0	0.1644	0.2708
34	RM3331	8.0	0.1191	0.2088
35	RM17	6.0	0.1540	0.2550
Mean		6.8	0.1702	0.2826

**Table 4:** Details of agronomic traits of Shanlan upland rice accessions.

No.	PH (cm)	PN	PL (cm)	TG	FG	SR (%)	TW (g)
M1	134.8	9.5	24.5	91.9	78.9	85.9	30.8
M2	147.8	8.4	25.6	94.6	85.7	90.6	27.5
M3	147.0	6.7	28.0	146.1	122.9	84.1	30.2
M4	154.0	8.2	26.3	126.4	116.6	92.2	29.0
M5	152.0	9.1	26.6	103.1	92.2	89.4	30.6
M6	136.2	8.4	25.9	102.5	87.9	85.8	30.3
M7	141.8	10.2	25.3	90.0	76.5	85.0	31.9
M8	156.6	12.6	28.3	103.5	85.9	83.0	33.2
M9	136.5	8.3	26.9	112.4	99.1	88.2	34.5
M10	148.7	9.7	25.8	87.3	69.4	79.5	28.2
M11	168.3	9.0	29.7	144.4	131.0	90.7	27.2
M12	164.0	11.7	26.3	137.0	121.8	88.9	31.4
M13	170.7	12.0	24.3	85.6	78.4	91.6	25.2
M14	175.0	11.0	25.9	104.3	84.6	81.1	28.7
M15	169.7	9.8	27.4	104.0	96.0	92.3	32.1
M16	156.3	10.3	29.4	140.9	105.9	75.2	30.6
M17	160.0	8.1	24.9	102.8	83.6	81.3	27.8
M18	137.3	6.0	29.0	95.2	61.0	64.1	24.2
M19	154.3	8.0	24.6	104.0	93.6	90.0	29.0
M20	159.7	8.0	28.6	108.6	95.4	87.8	27.4
M21	160.7	9.0	23.2	94.2	79.2	84.1	27.0
M22	144.3	7.0	26.6	133.0	104.4	78.5	28.0
M23	146.7	10.0	28.8	112.4	75.2	66.9	30.4
M24	151.0	8.0	29.0	115.2	97.2	84.4	29.0
M25	148.3	6.0	33.2	153.6	102.8	66.9	28.9
M26	152.7	7.0	30.2	124.0	75.4	60.8	29.0
M27	158.7	11.0	27.0	113.8	82.4	72.4	25.6
M28	160.7	9.0	27.8	131.0	70.0	53.4	28.0
M29	139.0	9.0	28.2	99.2	86.8	87.5	31.4
M30	155.7	7.0	27.2	195.8	131.4	67.1	24.2
M31	158.3	13.0	29.4	151.6	125.6	82.8	26.2
M32	162.7	11.0	27.4	118.8	92.6	77.9	30.0
M33	152.7	7.0	26.2	99.6	71.4	71.7	26.4
M34	162.0	11.0	29.2	144.6	129.8	89.8	25.8
M35	157.8	8.0	27.4	118.2	113.0	95.6	25.4
M36	135.8	14.0	28.3	196.0	162.0	82.7	17.7
M37	159.2	6.0	29.4	163.2	78.2	47.9	24.0
M38	143.2	7.0	28.7	170.6	107.0	62.7	30.6
M39	157.2	4.0	26.4	124.5	113.3	91.0	20.5
M40	153.1	5.0	31.4	189.2	166.4	87.9	23.6
M41	142.1	6.0	26.0	107.7	90.0	83.6	32.0
M42	156.1	5.0	27.3	174.8	151.2	86.5	31.4
M43	139.2	4.0	32.0	243.0	115.0	47.3	31.4
M44	149.5	5.0	24.8	141.8	101.4	71.5	30.4
M45	132.0	6.0	24.8	119.7	60.5	50.6	22.0
M46	145.0	5.0	26.5	184.2	148.2	80.5	34.8
M47	167.1	10.0	29.5	177.2	161.2	91.0	29.0
M48	139.2	9.0	29.3	180.0	157.4	87.4	31.2
M49	133.5	8.0	23.1	239.6	226.0	94.3	23.1
M50	157.5	6.0	31.3	182.7	111.3	60.9	33.5
M51	133.0	8.0	22.9	150.3	129.4	86.1	22.8
M52	147.5	4.0	34.1	188.5	107.0	56.8	24.0
M53	99.2	10.0	21.0	164.8	123.3	74.8	23.3
M54	123.0	7.0	25.1	205.9	153.3	74.5	23.2
M55	173.5	11.0	25.8	128.3	95.1	74.1	36.1
M56	86.3	5.0	19.0	84.2	67.6	80.3	21.0
M57	100.0	3.0	19.6	62.4	54.8	87.8	23.1
*P*	0.000	0.046	0.000	0.000	0.000	0.000	0.000

PH: plant height; PN: effective panicle number per plant; PL: panicle length; TG: total number of grains; FG: number of filled grains; SR: seed setting rate; TW: 1000-grain weight; *P*: levels of probability for significant differences between data within each column.

**Table 5:** Percentage of total variations explained by SSR markers linked to agronomic traits of Shanlan upland rice accessions (%).

No.	SSR marker	Agronomic traits						
		PH	PN	PL	TG	FG	SR	TW
1	OSR28	17.93		12.45				
2	RM17	10.20			11.40			24.11
3	RM190				9.50	10.42		
4	RM208	42.62		20.63	10.26			
5	RM21	10.28		16.56		23.19		
6	RM219	10.31						12.71
7	RM253	17.93		12.45	18.09		12.88	
8	RM267				9.71			
9	RM278							11.59
10	RM331					10.43		
11	RM332			12.36	22.52			
12	RM3331				9.54			
13	RM336		14.43					
14	RM423		15.81					
15	RM424	10.31						
16	RM481	28.67		12.43		19.16	13.55	
17	RM493	11.97	11.74	13.22	15.10	14.21	19.64	
18	RM567	17.93		12.45	11.95	24.61		
19	RM571						20.66	
20	RM583					0.12		
21	RM590	14.80						16.98
22	RM71	17.93		12.45				
23	RM7102							11.59
24	RM72		12.66		14.27			
25	RM85			24.18	9.44			

PH: plant height; PN: effective panicle number per plant; PL: panicle length; TG: total number of grains; FG: number of filled grains; SR: seed setting rate; TW: 1000-grain weight.

## Data Availability

Data used to support the findings of this study are available from the corresponding author upon request.
